# Comprehensive Assessment of Herbicide Toxicity on *Navicula* sp. Algae: Effects on Growth, Chlorophyll Content, Antioxidant System, and Lipid Metabolism

**DOI:** 10.3390/md22090387

**Published:** 2024-08-28

**Authors:** Chunyan Zheng, Jie Yang, Yunting Wang, Waqas Ahmed, Amir Khan, Jiannan Li, Jiechang Weng, Sajid Mehmood, Weidong Li

**Affiliations:** 1 College of Ecology, Hainan University, Haikou 570100, Chinaljn1998h@163.com (J.L.); 2 Center for Eco-Environment Restoration Engineering of Hainan Province, Hainan University, Haikou 570228, China; 3 Department of Medicine, Hainan Medical University, Haikou 571100, China; 4 Hainan Provincial Ecological and Environmental Monitoring Center, Haikou 570228, China

**Keywords:** herbicide exposure, *Navicula* sp., algae growth, antioxidant system, lipid metabolism

## Abstract

This study investigated the effects of herbicide exposure on *Navicula* sp. (MASCC-0035) algae, focusing on growth density, chlorophyll content, antioxidant system, and lipid metabolism. *Navicula* cultures were exposed to different concentrations of atrazine (ATZ), glyphosate (Gly), and acetochlor (ACT) for 96 h. Results showed a significant decrease in cell numbers, with higher herbicide concentrations having the most noticeable impacts. For instance, Gly-G2 had reduced cell populations by 21.00% at 96 h. Chlorophyll content varied, with Gly having a greater impact on chlorophyll a compared to ATZ and ACT. Herbicide exposure also affected the antioxidant system, altering levels of soluble sugar, soluble protein, and reactive oxygen species (ROS). Higher herbicide rates increased soluble sugar content (e.g., ATZ, Gly, and ACT-G2 had increased by 14.03%, 19.88%, and 19.83%, respectively, at 72 h) but decreased soluble protein content, notably in Gly-G2 by 11.40%, indicating cellular stress. Lipid metabolism analysis revealed complex responses, with changes in free proline, fatty acids, and lipase content, each herbicide exerting distinct effects. These findings highlight the multifaceted impacts of herbicide exposure on *Navicula* algae, emphasizing the need for further research to understand ecological implications and develop mitigation strategies for aquatic ecosystems.

## 1. Introduction

In recent years, the toxicity of herbicides to algae, which are key primary producers in ecosystems, and their ecotoxicological effects have gained increasing attention from researchers both locally and internationally [1,2]. The extensive and frequent use of herbicides poses potential risks to aquatic ecological environments due to the accumulation of their chemical residues in the soil and aquatic environments [3]. Herbicides are widely used in agricultural production and have a significantly greater toxic impact on algae compared to insecticides and fungicides [4]. Previous studies have reported that herbicide pollution in surface water has direct toxic effects on phytoplankton, epiphytes, and macroplant populations [5,6]. Herbicides can be classified according to their target location, mode of action, similarity of plant symptoms, or chemical class [7]. Atrazine, acetochlor, and glyphosate are typical examples of herbicides that belong to the triazine, amide, and organic phosphine classes. These three herbicides are utilized extensively in agricultural settings and are typically found in aquatic environments [8,9,10].

Atrazine, often known as ATZ, is a synthetic triazine herbicide [11]. ATZ is one of the most extensively used herbicide kinds and has held a significant place in the TP herbicide industry for many years, as the largest variety of TP herbicides [10]. China and the US have set a maximum detection limit of 3 µg L^−1^ for ATZ in aquatic environments [12]. Acetochlor, often known as ACT, is a chloroacetanilide herbicide. It is frequently found in freshwater habitats because of its strong stability and ability to dissolve in water [13]. ACT, a substance of concern, is present in both surface and drinking water during the spring and fall seasons in the United States. It is identified as one of the primary factors contributing to potential ecological hazards in the Midwest watershed, as stated by Li et al. (2018) [14]. Both alachlor and acetochlor have been categorized as B-2 carcinogens by the US Environmental Protection Agency (EPA). Additionally, the European Commission (EC) has chosen not to register acetochlor [15]. Glyphosate, also known as N-(methyl phosphate) glycine or Gly, is the most extensively utilized herbicide globally. Its widespread adoption is attributed to its exceptional effectiveness, minimal toxicity, and ability to target a wide range of plant species. Glyphosate consistently holds the top position in terms of annual sales value among all herbicides [16]. Glyphosate, when introduced into water, hampers the growth of algae by means of phytotoxicity. Additionally, its byproduct, aminomethylphosphonic acid (AMPA), may pose greater environmental hazards [17]. Furthermore, research has demonstrated that glyphosate at a concentration of 2.5 mg L^−1^ has a notable impact on gene transcription in specific cyanobacteria, as reported by Lu et al. in 2020 [18]. Upon the application of Gly, there will be a loss of 30% that will enter the aquatic environment. Therefore, it is crucial to focus on the impact of Gly on water environment quality [19].

Diatoms are unicellular eukaryotic organisms that have the ability to carry out photosynthesis on their own. According to Falciatore et al. (2020) [20], their presence on Earth dates back 165 million years. They are a significant primary source of water, accounting for approximately 20% of the primary productivity of the entire ocean [21], and have a crucial role in the global silicon cycle. Diatoms have gained significant attention both domestically and internationally as a potential raw material for biodiesel synthesis. This is due to their rapid growth rate, short growth cycle, and high oil content. Diatoms are considered the third generation of biomass energy. Diatoms are prevalent in diverse natural habitats, including seawater, rivers, ponds, and industrial wastewater. They possess the ability to sustain their stability in different conditions, such as varying acidity levels, high hydrochloric acidity, elevated salinity, contamination by heavy metals, and the eutrophication of lakes resulting from natural or human-induced processes [22]. *Navicula* sp., often known as *Navicula*, is the most diversified genus within the diatom phylum [23,24]. It is frequently used as a standard microalga for assessing the toxicity of pollutants in water [25]. It possesses several attributes, including a high level of photosynthetic efficiency, rapid reproductive capabilities, and robust environmental adaptation. The proliferation of diatoms in an aquatic setting is significantly impacted by the microenvironment of the water. Specifically, herbicides have targeted effects on benthic diatoms, causing considerable changes in the establishment of communities and impacting benthic habitats and ecosystem function [26]. Hence, the assessment of the diatom index in a water body enables a focused evaluation of the present microenvironmental condition of the water [27]. By examining the response mechanism of algae to herbicides like ATZ, Gly and ACT, we can gain a deeper understanding of how aquatic organisms adapt to polluted settings. This knowledge can help us forecast the future trajectory of aquatic ecosystems. Furthermore, this research offers a scientific foundation for implementing measures to limit water pollution and provides fundamental data for future toxicity studies on benthic diatoms and their utilization in biological monitoring.

## 2. Results

### 2.1. Effect of Herbicides on the Growth Density of Navicula

**Figure 1:**
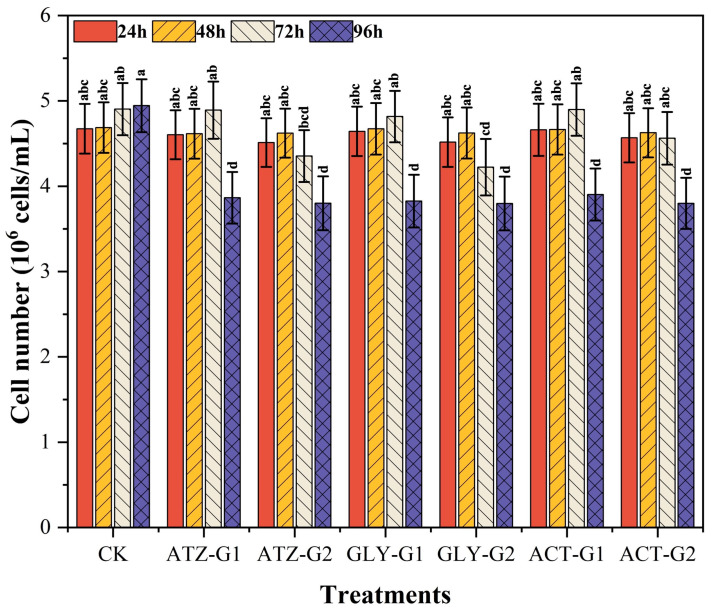
Cell numbers of *Navicula* after 96 h of exposure to herbicides. Different letters indicate significant differences (*p* < 0.05). Bars are means ± SD (n = 3).

Exposure to all herbicides had negative impact on the density of *Navicula*, as shown in Figure 1. The initial density of Navicula was 4.6 × 10^6^ cells/mL. The results indicate that a substantial dosage of herbicides resulted in a notable reduction in the quantity of cells (Figure 1). After a period of 96 h, the presence of Gly had a significant effect on the number of cells at all concentrations and resulted in a drop of 9.22% in G1 and 21.00% in G2 compared to the control. Within the initial 24 h period, a consistent trend was noticed, wherein ATZ-G2, Gly-G2, and ACT-G2 resulted in a notable reduction in cell counts by 3.45%, 10.75%, and 2.26%, respectively, compared to the control group. Continued exposure to higher medication dosages at 48 and 72 h led to reductions in cell counts. Specifically, there was decrease of 3.52% and 9.16% in ATZ-G2, 15.06% and 15.12% in Gly-G2, and 5.56% and 2.88% in ACT-G2, respectively, compared to the control.

### 2.2. Effect of Herbicides on the Chlorophyll Content of Navicula

**Figure 2:**
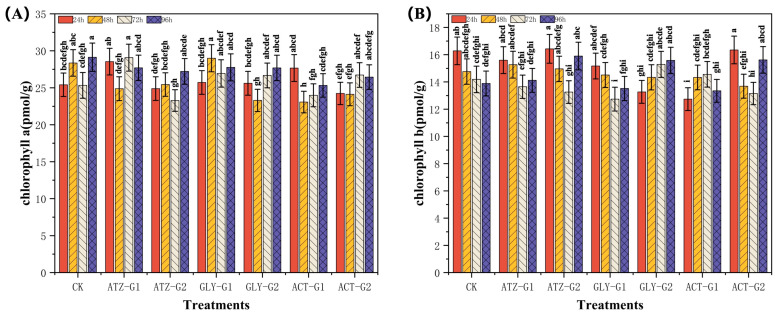
Photosynthetic pigment content in *Navicula* after 96 h of exposure to herbicides. (**A**) Chlorophyll a, (**B**) Chlorophyll b. Different letters indicate significant differences (*p* < 0.05). Bars are means ± SD (n = 3).

Chlorophyll content, the main pigment involved in photosynthesis, directly reflects the growth status and photosynthetic efficiency of algae. This study observed significant variations in the chlorophyll levels of *Navicula* under different herbicide treatments. Figure 2A,B illustrates the variations in photosynthetic pigment levels in the boat-shaped algae. At a concentration of 100 µg L^−1^, the chlorophyll a content of *Navicula* in the ATZ-G1 phase showed a considerable increase at 24 h, followed by a decline. By 48 h, it had reached the lowest level compared to the control group, with a decrease of 12.3% (Figure 2A). However, unlike ATZ-G1, the chlorophyll a content of ACT-G1 had first increased by 8.9% at 24 h and then had rapidly declined by 18.7% at 48 h compared to the control group. The Gly-G1 was decreased by 4.7 units throughout a 96 h period. When compared to the control group, the ATZ-G2, Gly-G2, and ACT-G2 groups exhibited a general drop in chlorophyll a content throughout the culture period. Specifically, at 48 h, the chlorophyll a content had declined by 10.3%, 17.8%, and 15.0% in the ATZ-G2, Gly-G2, and ACT-G2 groups, respectively (Figure 2A).

The Gly group exhibited the most significant changes in chlorophyll a level compared to the control group. After 24 h, chlorophyll a concentration in Gly-G1 had decreased by 6.8%, while in Gly-G2, it had dropped by 18.5%. Increasing the concentration of Gly has a proportional effect on the chlorophyll-B content in *Navicula* algae, as shown in Figure 2B. ATZ-G1 exhibited a reduction of 4.2% after 24 h, however ATZ-G2 did not display any noteworthy deviation when compared to the control group. ACT-G1 and ACT-G2 exhibited contrasting patterns in chlorophyll b levels after 24 h. ACT-G1 had a substantial decline of 21.7%, whereas ACT-G2 did not demonstrate any significant change compared to the control group.

### 2.3. Effect of Herbicides on the Antioxidant Activities of Navicula

**Figure 3:**
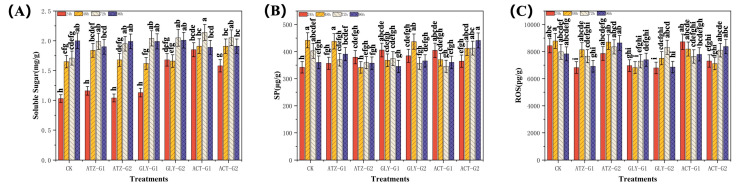
Soluble sugar (SS; (**A**)), soluble protein (SP; (**B**)) and reactive oxygen species (ROS; (**C**)) in *Navicula* after 96 h of exposure to herbicides. Different letters indicate significant differences (*p* < 0.05). Bars are means ± SD (n = 3).

According to Figure 3A, the amount of soluble sugar (mg g^−1^) in *Navicula* rose to varying extents when exposed to different doses of atrazine (ATZ), glyphosate (Gly), and acetochlor (ACT). After 72 h, the levels of soluble sugar were considerably higher in all treatment groups compared to the control group. The treatments ATZ-G1 and ATZ-G2 showed a rise of 16.4% and 14.0%, respectively. Gly-G1 and Gly-G2 exhibited an increase of 19.3% and 19.9%, respectively. ACT-G1 and ACT-G2 demonstrated an increase of 25.1% and 19.9%, respectively. The increase observed was strongly correlated with the duration of exposure, and the concentration of soluble sugars in *Navicula* showed a substantial rise as the exposure time rose. Subsequently, there was a decline in the soluble sugar level over a period of 96 h in all treatment groups as the exposure time increased. However, this decline was not statistically significant when compared to the control group. These findings indicate that extended exposure to ATZ, Gly, and ACT can result in a substantial rise in the number of soluble sugars in *Navicula*, particularly at higher doses and longer durations of exposure.

Figure 3B demonstrates that the concentration of atrazine, glyphosate, and acetochlor had varied effects on the soluble protein (SP) content (pg g^−1^) of *Navicula*, causing a reduction. As the exposure period increased, the concentration of SP in *Navicula* fell considerably. In the treatment group exposed to ATZ, the soluble protein content of ATZ-G1 did not show a significant reduction at 48 h compared to the control group. However, the soluble protein content of ATZ-G2 had decreased by 22.7% at 48 h and continued to decline as the exposure time increased. Both the Gly group and the ACT group exhibited a pattern of initial growth followed by subsequent decline. In the Gly treatment group, the soluble protein content in 48 h Gly-G1 and Gly-G2 exhibited a pattern of initial increase followed by reduction when compared to the control group. Specifically, at 48 h, the soluble protein content had increased by 18.7% and 12.4% in 48 h Gly-G1 and Gly-G2, respectively. Nevertheless, as the duration of exposure increased, the group treated with Gly exhibited a notable downward trend in comparison to the control group. The most pronounced drop, amounting to 16.6%, was observed in GLY-G1 after 72 h. In the ACT therapy group, the levels of ACT-G1 and ACT-G2 had increased by 18.3% and 6.7% after 48 h, respectively. However, at 72 h, there was a decline in both ACT-G1 and ACT-G2, with a reduction of 16.1% and 6.6%, respectively. Among these, ACT-G1 had the most significant impact on the soluble protein content of *Navicula*. The findings indicate that extended exposure to ATZ, Gly, and ACT can cause substantial declines in *Navicula* SP levels, particularly at higher concentrations and longer durations of exposure.

Figure 3C demonstrates that the levels of reactive oxygen species (ROS) in *Navicula* had dropped to different extents after being exposed to atrazine, glyphosate, and acetochlor at G1 and G2 levels for 24 h, 48 h, 72 h, and 96 h. The group treated with ATZ showed a drop in reactive oxygen species (ROS) levels of ATZ-G1 and ATZ-G2 in *Navicula* after 24 h, with reductions of 19.1% and 6.8%, respectively. Nevertheless, when the exposure time was extended, the levels of ATZ-G1 and ATZ-G2 ROS exhibited contrasting changes at 72 h in comparison to the control group. ATZ-G1 fell by 4.0%, but ATZ-G2 increased by 4.6%. In comparison to the control group, the group treated with Gly exhibited a noticeable decline. The most significant decrease in GLY-G1 and GLY-G2 was observed at 48 h, resulting in a reduction of 22.3% and 14.5% in the ROS of *Navicula*, respectively. In the ACT treatment group, the ACT-G1 exhibited a gradual rise of 3.5% at the 24 h mark; however, this difference did not reach statistical significance when compared to the control group. Conversely, the ACT-G2 displayed a declining pattern at 24 h, resulting in a 13.3% drop in the reactive oxygen species (ROS) in *Navicula*. Surprisingly, when the exposure duration was extended, the concentration of ATZ and ACT in 96 h ROS at high doses (300 µg L^−1^) rose compared to the control group. Specifically, ATZ-G2 increased by 10.3% and ACT-G2 increased by 6.9%. The data indicate that extended exposure to ATZ, Gly, and ACT can cause a substantial decrease in *Navicula* ROS, particularly at elevated concentrations and prolonged durations of exposure.

**Figure 4:**
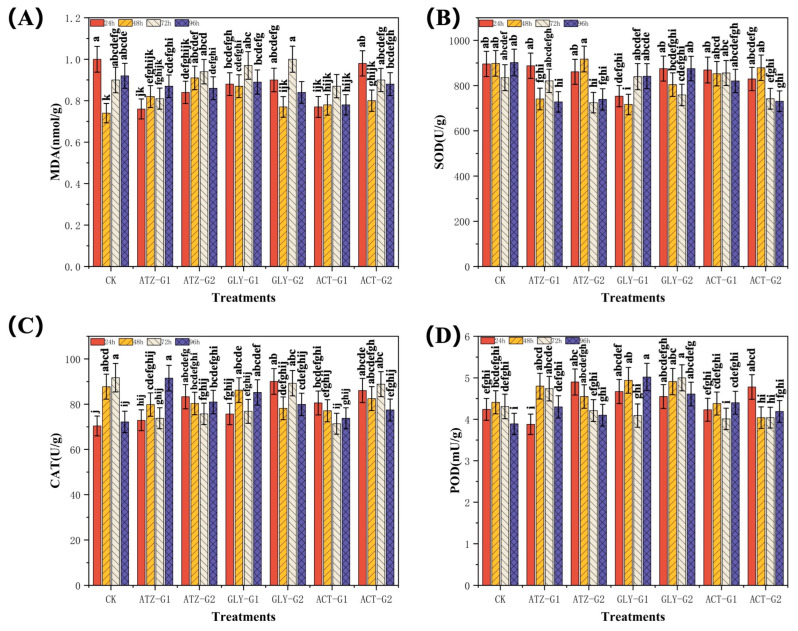
Malondialdehyde (MDA; (**A**)), superoxide dismutase (SOD; (**B**)), catalase (CAT; (**C**)) and peroxidase (POD; (**D**)) in *Navicula* after 96 h of exposure to herbicides. Different letters indicate significant differences (*p* < 0.05). Bars are means ± SD (n = 3).

Figure 4A demonstrated a significant change in the content of malondialdehyde (MDA) (nmol g^−1^) in *Navicula* when exposed to atrazine (ATZ), glyphosate (Gly) and acetochlor (ACT) at a concentration of 100 µg L^−1^ and 300 µg L^−1^. *Navicula* exhibited a declining trend in MDA content at both 24 h and 96 h intervals in comparison to the control group. ATZ-G1 and ATZ-G2 had fallen by 24.0% and 16.0%, respectively. Gly-G1 and Gly-G2 had declined by 10.0% and 23.0%, respectively. ACT-G1 and ACT-G2 had reduced by 23.0% and 2.0%, respectively. Nevertheless, when the concentrations were elevated and the exposure intervals were extended, the rise in MDA content surpassed that of the control group to a greater extent. At the 48 h mark, the concentration of MDA in *Navicula* had increased by 10.8% and 23.0% under the ATZ-G1 and ATZ-G2 treatments, respectively. Long-term exposure to high concentrations of ATZ is likely to result in damage to the cell membranes of *Navicula*, leading to a rise in MDA content. After 72 h, the MDA levels of Gly-G1 and Gly-G2 had increased by 7.8% and 11.1%, respectively. However, after 96 h, the MDA levels of Gly-G1 and Gly-G2 had reduced by 3.3% and 8.7%, respectively. Overall, the concentration of MDA in *Navicula* decreased when treated with Gly. Simultaneously, the levels of MDA in *Navicula* exposed to ACT-G1 and ACT-G2 reduced by 15.2% and 4.3%, respectively, within a 96 h period. The reduction in MDA content was most pronounced in the ACT-G1 treatment. The concentration of MDA in Navicula varies when exposed to different herbicides. ATZ and Gly can potentially cause increased cell membrane damage, although MDA levels tend to decrease after initially rising. On the other hand, ACT can induce cell membrane damage within a brief timeframe. Nevertheless, algal cells have the ability to mitigate the harm by employing their own reparative system throughout extended periods of exposure. These findings enhance our comprehension of the possible ecological hazards that these three herbicides pose to aquatic life.

Figure 4B demonstrates that exposure to atrazine (ATZ), glyphosate (Gly), and acetochlor (ACT) at G1 (100 µg L^−1^) and G2 (300 µg L^−1^) concentrations significantly decreased the superoxide dismutase (SOD) activity of *Navicula* compared to the control group. In the group treated with ATZ, the level of 48 h ATZ-G1 had reduced by 17.5%, whereas ATZ-G2 had raised the activity of SOD by 2.1% after 48 h. This change in SOD activity may be associated with the early stage of exposure to the herbicide, which is triggered by a reaction to oxidative stress. Nevertheless, as the exposure duration increased, the SOD activity of ATZ-G1 and ATZ-G2 rapidly declined, decreasing by 19.3% and 18.1% correspondingly after 96 h. Within the Gly group, the SOD activity of Gly-G1 saw a substantial decline of 16.0% after 24 h; however, the SOD activity of Gly-G2 had only decreased by 3% compared to the control group, which was not a significant reduction. Nevertheless, after 48 h, the superoxide dismutase (SOD) activity of both Gly-G1 and Gly-G2 had declined by 20.2% and 10.5%, respectively. This decline could be attributed to the initial weak impact of Gly on the algal cells. ACT-G1 and ACT-G2 exhibited a general decline in SOD activity throughout the exposure period, in contrast to ATZ and Gly. While the SOD activity of ACT-G1 and ACT-G2 showed opposite changes at 72 h, ACT-G1 grew by 2.5% while ACT-G2 declined by 11.2%. However, the rise in ACT-G1 at 72 h was not statistically significant when compared to the control group. As the duration of exposure increased, the levels of 96 h superoxide dismutase (96 h SOD) in ACT-G1 and ACT-G2 fell by 9.0% and 19.0% correspondingly. These findings indicate that the impacts of atrazine, glyphosate, and acetochlor on the oxidative stress of *Navicula* are governed by distinct pathways and exhibit unique properties. Prolonged exposure to these herbicides can result in different levels of harm or suppression of the antioxidant system in algal cells, consequently impacting the growth and metabolism of these cells.

Figure 4C demonstrates a considerable alteration in the activity of *Navicula* catalase (CAT) (U g^−1^) after being exposed to atrazine (ATZ), glyphosate (Gly), and acetochlor (ACT) at G1 and G2 levels. In general, when compared to the control group, the activity of CAT initially increased at the start of exposure, then steadily declined, and then increased once more. When comparing the CK group to both ATZ-G1 and ATZ-G2, it was found that both ATZ-G1 and ATZ-G2 had increased CAT activity after 24 h. Specifically, ATZ-G1 had raised CAT activity by 3.6% and ATZ-G2 had increased by 18.4%. These findings indicate that atrazine exposure likely caused oxidative stress in the algal cells, resulting in increased CAT activity. Nevertheless, as the duration of exposure grew, the catalase (CAT) activity of ATZ-G1 exhibited a progressive rise after reaching its lowest point at 72 h. At 96 h, the CAT activity of AZT-G1 had reached its peak at 26.8%, while the CAT activity of ATZ-G1 at 96 h had increased by 12.2% compared to the control group. The impact of Gly on *Navicula* CAT activity was comparable to that of the ATZ treatment group. The catalase (CAT) activity of Gly-G1 and Gly-G2 showed an increase after 24 h. Specifically, the CAT activity of Gly-G1 had grown by 7.5% and the CAT activity of Gly-G2 had increased by 28.0% after 24 h. After 48 h, Gly-G1 and Gly-G2 had caused a reduction in CAT activity in *Navicula*. Specifically, Gly-G1 led to a decrease of 1.6%, while Gly-G2 resulted in a fall of 10.9%. By extending the duration of exposure, the concentration of CAT in *Navicula* increased once more after 96 h. These findings indicate that glyphosate’s impact on oxidative stress in *Navicula* is gradual, and as the exposure duration increases, the algae cells gradually adjust and raise their catalase (CAT) activity to manage oxidative stress. During ACT administration, both ACT-G1 and ACT-G2 exhibited elevated CAT activity after 24 h. However, the enzyme activity steadily declined as the exposure period rose. ACT-G1 and ACT-G2 exhibited a 14.4% and 22.3% increase at 24 h, respectively, followed by a quick decline of 22.2% and 3.1% at 72 h. To summarize, atrazine, glyphosate, and ethephon have distinct impacts on the catalase activity of *Navicula*.

Figure 4D demonstrates that the peroxidase (POD) activity of *Navicula* rose significantly after being exposed to atrazine (ATZ), glyphosate (Gly), and acetochlor (ACT) at G1 and G2 levels. During the initial phase of exposure, the activity of POD (peroxidase) primarily exhibited an increase, potentially due to the presence of oxidative stress induced by exposure to herbicides. Nevertheless, as the duration of exposure increased, the alteration in the trend of POD activity differed depending on the kind and concentration of the herbicide. In the group treated with ATZ, there was a decrease of 8.5% in ATZ-G1 levels at 24 h compared to the control group. However, the levels grew again and reached their highest point at 96 h, with a rise of 10.5%. Nevertheless, when subjected to ATZ-G2 treatment, the peroxidase (POD) activity of *Navicula* first exhibited an upward trend, with a notable increase of 15.6%. However, it subsequently declined, reaching its lowest level at 72 h, with a loss of 2.3%. In the Gly treatment group, the peroxidase (POD) activity of both GlY-G1 and Gly-G2 initially rose upon exposure, but later exhibited distinct patterns. The post-oxidative damage (POD) activity of Gly-G1 had reached its lowest level (5.1%) after 72 h and subsequently grew to its maximum at 96 h, exhibiting a 29.0% increase compared to the control group. Nevertheless, when subjected to Gly-G2 therapy, the peroxidase (POD) activity of *Navicula* continued to rise, ultimately peaking after 96 h, exhibiting a notable increase of 18.5%. ACT showed a smaller impact on the POD activity of *Navicula* compared to ATZ and Gly. When comparing the control group to the group of algae treated with ACT-G1, the activity of POD (peroxidase) continuously dropped during the exposure period. It had reached its lowest value at 72 h, showing a fall of 6.7%. After that, there was a gradual increase of 13.1% up to 96 h. On the other hand, the value of ACT-G2 had risen by 12.7% after 24 h and then had declined by 8.4% after 48 h. The results demonstrated that ATZ, Gly, and ACT had distinct impacts on the peroxidase activity of *Navicula*. Prolonged exposure to these herbicides can result in different levels of harm or suppression of the antioxidant system in algal cells, hence impacting the growth and metabolism of the cells.

### 2.4. Effect of Herbicides on the Lipid Composition of Navicula

**Figure 5:**
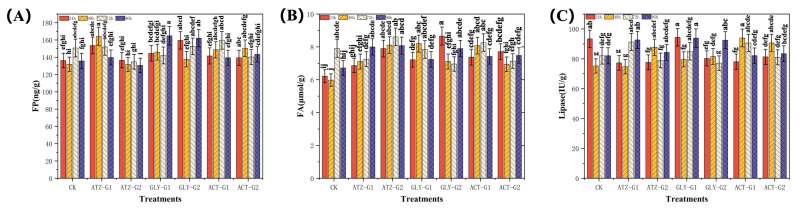
Free proline (FP; (**A**)), fatty acid (FA; (**B**)) and lipase (**C**) in *Navicula* after 96 h of exposure to herbicides. Different letters indicate significant differences (*p* < 0.05). Bars are means ± SD (n = 3).

Figure 5A–C display the levels of free proline (FP), fatty acid (FA), and lipase in *Navicula*. The concentration of FP (ng g^−1^) in *Navicula* is seen to increase in the presence of ATZ, Gly, and ACT. Figure 5A. The *Navicula* FP of ATZ, Gly, and ACT exhibited a rising pattern, as depicted in Figure 5A. The *Navicula* samples in the ATZ treatment group exhibited varying trends in FP content. Specifically, the FP content of *Navicula* treated with AZT-G1 for 48 h increased by 24.5%. However, the change in FP content of *Navicula* treated with ATZ-G2 for 48 h was not statistically significant when compared to the control group. As the exposure period increased, the FP content of the ATZ-G2 therapy reduced steadily. The ATZ-G2 experienced a significant decline, reaching its lowest point in the last 72 h with a decrease of 10.6%. In the group treated with Gly, the fatty acid content of *Navicula* mostly exhibited an upward trend. The fractional protein (FP) content of Gly-G1 and Gly-G2 had grown by 6.1% and 17.1%, respectively, after 24 h. However, Gly-G1 exhibited a decreasing trend at 72 h and subsequently increased by 5.7%. This fluctuation in Gly-G1’s FP content may be attributed to the algal stress response. In the group receiving ACT treatment, the FP content at 48 h in *Navicula* had grown to its highest level, with ACT-G1 and ACT-G2 increasing by 12.7% and 14.0%, respectively. The presence of varying quantities of herbicide resulted in distinct levels of free proline accumulation in *Navicula*, indicating that the algae may counteract the herbicide’s effects by accumulating free proline.

Figure 5B demonstrates that the fatty acid (FA) content (μmol g^−1^) of *Navicula* exhibited distinct patterns following exposure to atrazine, glyphosate, and acetamiprid at G1 and G2 levels. The fatty acid (FA) content of *Navicula* exhibited a notable rise as the exposure duration was extended. After 24 h, the application of ATZ-G1 and ATZ-G2 had resulted in a considerable increase in FP activity compared to the control group. Specifically, ATZ-G1 had increased by 10.5% and ATZ-G2 had increased by 27.0%. Nevertheless, ATZ-G1 saw a significant decline, reaching its minimum value after 72 h, with a decrease of 8.2%. During Gly treatment, the levels of Gly-G1 and Gly-G2 had increased by 16.0% and 39.2%, respectively, after 24 h. However, after 72 h, there was a drop in Gly-G1 and Gly-G2 levels, with a fall of 0.8% and 11.9%, respectively. In the group receiving ACT treatment, the frequency of *Navicula* generally exhibited a rising trend. Specifically, ACT-G1 and ACT-G2 rose by 35.1% and 16.1%, respectively, over a 48 h period. When the stress time was extended, the FP content of ACT-G2 had reduced by 9.6% after 72 h in comparison to ACT-G1. This shift was particularly noticeable in the simultaneous treatment shown in Figure 5B.

Figure 5C demonstrates that the lipase content (IU g^−1^) of *Navicula* dropped to different extents in the initial phases following treatment with atrazine, glyphosate, and acetochlor at G1 (100 µg L^−1^) and G2 (300 µg L^−1^) concentrations, respectively. At 24 h, the lipase activity of *Navicula* had reduced by 17.2% and 16.8% with ATZ-G1 and ATZ-G2 therapy, 16.4% and 12.8% under ACT-G1 and ACT-G treatment, and 14.0% by Gly-G2 treatment compared to the control group. Although the lipase content following Gly-G1 therapy increased by 1.1%, there was no statistically significant difference compared to the control group. After 96 h, the lipase content of ATZ-G1 had increased by 12.9% compared to the control group. However, there was no significant difference in the rising trend of Gly-G1 and Gly-G2 at 96 h, as they climbed by 14.4% and 12.7%, respectively. In the ACT group, the concentration of lipase had fallen after 24 h and then had increased again after 48 h. Additionally, the concentrations of ACT-G1 and ACT-G2 had increased by 24.9% and 20.4%, respectively, after 24 h. These findings indicate that the impact of long-term exposure to ATZ, Gly, and ACT on the lipase content of *Navicula* is dependent on both the duration of exposure and the concentration of the substances.

## 3. Discussion

Herbicides, a major category of pesticides, have the potential to affect the structure and function of aquatic ecosystems both directly and indirectly [28]. This is because they are commonly found in agricultural land and surface water [29]. The contamination of herbicides presents a worldwide peril to both vegetation and freshwater ecosystems [30]. Algae serve as primary producers in aquatic ecosystems and serve as significant indicators of water quality [31]. They play a crucial role in evaluating the condition of aquatic ecosystems and monitoring the presence of environmental contamination. Microalgae have a significant impact on the worldwide carbon cycle and global climate. Understanding how microalgae respond to various environmental conditions and changes in nutrient levels in wastewater is crucial. Herbicides are the predominant organic pollutants found in groundwater [32]. Despite their low levels in water, they can have a significant impact on the growth of various organisms even at low concentrations [33]. The growth inhibition assay of *Navicula* was conducted to examine the toxicity of atrazine (ATZ), glyphosate (Gly), and acetochlor (ACT). The assessment of toxic effects often involves evaluating alterations in growth inhibition and enzyme activity in herbicide cultures compared to herbicide-free control cultures [1,34]. Reaction of algae to herbicides can differ significantly based on factors such as the specific species of algae, the concentration of the herbicide, and the mechanism of action of the herbicide [35]. Given the widespread presence of herbicides in surface waters, it is crucial to investigate their toxicological impacts on primary producers and gain insights into interspecies variations. This knowledge may offer strategies for more effectively controlling the utilization of these herbicides in order to safeguard aquatic environments. A study by Dupraz et al. (2016) [36] investigates the immediate harmful effects of herbicides on microalgae. Microalgae exhibit varying levels of sensitivity when exposed to ATZ, regardless of their categorization [30]. ATZ in the water column can harm non-target organisms, such as freshwater algae, causing significant negative consequences [37]. ATZ suppresses the growth of *Chlamydomonas reinhardtii* and also causes a rise in carbohydrate content and the buildup of chlorophyll a [38]. DeLorenzo et al. (2004) [2] showed that atrazine concentrations of 12.5 µg L^−1^ or higher resulted in notable decreases in cell density, productivity, biomass, and biovolume across all algal groups. Algae and terrestrial plants share similar physiological and biochemical characteristics, indicating that algae are very vulnerable to Gly, as stated by Annett et al. (2014) [39]. The concentration of 0.125 mg L^−1^ of Gly has been found to enhance the primary productivity of phytoplankton, as demonstrated by Schaffer and Sebetich (2004) [40]. Exposure to high quantities of Gly herbicide (8.0 mg L^−1^) in freshwater environments can cause eutrophication of water bodies. This occurs because Gly leads to the generation of total phosphorus, which encourages the growth of periphyton in cyanobacteria [41]. According to Romero et al. (2011) [42], the MDA concentration of *Chlorella vulgaris* increased significantly when exposed to various doses of Gly, suggesting damage to lipid membranes.

Herbicides primarily exerts their harmful effects by suppressing cell division and protein synthesis, which has a detrimental impact on the growth and reproduction of aquatic species [43]. Consistent with prior research, our present data indicate that the use of atrazine (ATZ), glyphosate (Gly), and acetochlor (ACT) causes oxidative harm to the lipids of *Navicula*. Apoptosis can be triggered in surviving cells when the level of oxidative damage reaches beyond a certain threshold. Prior research has demonstrated that elevated levels of herbicides impede the growth of phytoplankton [44], whereas lower amounts facilitate phytoplankton growth by either releasing or stimulating nutrients [45]. The growth of *Navicula* seemed to be influenced solely by the high treatment and not the low treatment (Figure 1). Nevertheless, *Navicula* exhibited varied decreases in soluble protein, reactive oxygen species, and superoxide dismutase (Figure 3A,C and Figure 4B), indicating a compromised antioxidant system. Consequently, this led to a decline in photosynthetic performance. The herbicides had a considerable impact on the photosynthetic system of *Navicula*, causing notable changes in the biological processes linked to fatty acid metabolism (Figure 5B,C). These findings indicate that the three herbicide pesticides have harmful impacts on the development and antioxidant system of *Navicula*.

Chlorophyll is a pigment widely found in planktonic algae [46]. Its content can objectively reflect plant growth and photosynthesis levels and is used as an important indicator of algal response to pollutants [47]. Herbicides hinder photosynthesis by impeding the synthesis of photosynthetic pigments in algae. ATZ directly impacts the growth of microalgae by reducing photosynthesis through its action on photosystem II [48]. Both targeted and non-targeted plants that are exposed to atrazine commonly experience oxidative stress as a result of the generation of reactive oxygen species, such as superoxide anion radicals, hydroxyl radicals, mono-linear oxygen, and hydrogen peroxide [49]. Nevertheless, the precise mechanism by which Gly and ACT exert their harmful effects may be more intricate. Gly, a herbicide belonging to the organophosphorus class, disrupts the neurological system of organisms by principally inhibiting the action of acetylcholinesterase, which affects neurotransmission [50]. Over a period of 24 h, exposure to Gly at hazardous levels resulted in a decline in both the pigment content and the maximal quantum efficiency of Chlorella photosystem II (Fv/Fm), with the drop becoming more pronounced as the concentration of Gly increased [51]. Furthermore, the presence of herbicides led to a notable reduction in chlorophyll a and chlorophyll b, which are the primary pigments involved in the photosynthetic process (Figure 2B,C). The decrease in chlorophyll content indicates that the exposure to herbicides has an impact on the algae’s capacity to produce chlorophyll or components of the reaction center complex.

The chlorophyll content in the group treated with ATZ exhibited a declining pattern as the exposure period increased. Specifically, when exposed to a high concentration (300 μg L^−1^) of ATZ, the chlorophyll b content in *Navicula* fell by around 19.3% within 72 h. This indicates that atrazine had a suppressive impact on the photosynthesis of *Navicula*, as shown in Figure 2B. On the other hand, the Gly-treated group experienced a decline in chlorophyll content during the initial stage (24–72 h), followed by a subsequent upward trend. The early inhibitory effect of Gly on algal development may explain this phenomenon. However, as the exposure duration extended, the algae may have reacted to the environmental stress by modifying their physiological systems, resulting in a rebound in chlorophyll concentration (Figure 2A). The alterations in chlorophyll content in the group treated with ACT were more intricate. When exposed to a low concentration (100 μg L^−1^) treatment, the chlorophyll a content initially declined to its lowest value after 48 h and then climbed. On the other hand, under the high dosage (300 μg L^−1^) treatment, the chlorophyll content showed a steady increase (Figure 2A). These findings indicate that the impact of ACT on photosynthesis in *Navicula* is likely influenced by the concentration and duration of treatment.

Proteins have significant roles in the physiological and structural aspects of plant life, and various environmental conditions or pressures can exert distinct impacts on protein metabolism [52]. The primary factor leading to toxicity in algae from external sources is the excessive presence of reactive oxygen species (ROS), which induces oxidative stress [53]. Antioxidant components, including superoxide dismutase (SOD), catalase (CAT), and peroxidase (POD), are recognized as reliable markers for assessing the levels of oxidative stress. Prior research has demonstrated that numerous contaminants induce the generation of reactive oxygen species (ROS) within cells [54]. Elevated levels of reactive oxygen species (ROS) induce oxidative harm to proteins and lipids, ultimately resulting in damage to various organelles. Various antioxidant processes are also initiated to avert harm to proteins and lipids. Specific reactions can also result in heightened activity of superoxide dismutase, catalase, and peroxidase. *Navicula* can undergo substantial alterations in its physiological and biochemical characteristics as a result of extended exposure to ATZ, Gly and ACT. The dose and duration of exposure to the substance resulted in a considerable rise in soluble carbohydrates and reactive oxygen species content (Figure 3A,C). Conversely, there was a significant decrease in soluble protein and free proline content (Figure 3B and Figure 5B).

When exposed to low doses (0–100 μg L^−1^) of ATZ, Gly and ACT, *Navicula* experienced minor growth modifications that did not lead to significant alterations in antioxidant enzyme activity (Figure 3). Nevertheless, the application of herbicide treatments at concentrations ranging from 100 to 300 μg L^−1^ resulted in oxidative stress. When exposed to Gly stress at a concentration below 100 μg L^−1^, the activity of SOD increased, whereas the activities of CAT and POD did not alter (Figure 4). This indicates that SOD plays a crucial role as the primary antioxidant in the cellular defense against oxidative stress. On the other hand, CAT and POD may not be the most effective methods for cellular defense against toxic stress. The treatment with 100 μg L^−1^ Gly had a suppressive impact on photosynthetic activity. The occurrence of photosynthesis is also linked to the generation of reactive oxygen species, which are secondary products formed when the flow of electrons in photosynthesis is impeded [55]. Therefore, the rise in SOD activity plays a crucial function in preventing the removal of reactive oxygen species (ROS) caused by photosynthesis. The activity of POD initially increased and thereafter declined (Figure 4D). Furthermore, the expression of CAT increased even more after being exposed to 300 μg L^−1^ of ATZ for 96 h (Figure 4C). These data indicate that the therapy with the highest dose resulted in a stronger activation of antioxidant defense. However, it did not effectively address the progressive buildup of reactive oxygen species (ROS), which could potentially impair the long-term protective mechanisms of the antioxidant system. Impairment of the antioxidant defense system results in irreversible harm to cell membranes and malfunctioning of cells. An imbalance in the levels of antioxidant components and the production of reactive oxygen species (ROS) might initiate the process of membrane lipid peroxidation, resulting in the buildup of malondialdehyde (MDA) [56]. The concentration of MDA, a prevalent byproduct of lipid peroxidation, could indicate the degree of cellular membrane impairment [57]. The MDA content showed a more pronounced rise in both concentration and duration of exposure compared to the control group. The decline in MDA levels as exposure duration increases suggests that oxidative damage diminishes due to the activation of antioxidant defenses (Figure 4A). The alterations in MDA, SOD, CAT, POD, and ROS levels demonstrated that ATZ, Gly and ACT stimulated the antioxidant defense mechanisms in *Navicula*. Furthermore, these results exhibited variation in accordance with the duration of exposure, suggesting a time-dependent reaction by the antioxidants. It has been observed that herbicides have a comparable impact on the antioxidant defense of *Chlorella vulgaris*, as reported by Jiao et al. (2022) [58]. Therefore, the various alterations in the antioxidant system in *Navicula* due to herbicide-induced oxidative stress indicate that *Navicula* triggers distinct regulatory pathways and adaptive mechanisms in response to such stress.

Diatoms have gained recognition as a promising feedstock for biodiesel production in recent years [59]. This is primarily due to their rapid growth rate, short development cycle, and high oil content. Consequently, they are widely acknowledged as the third generation of biomass energy, both nationally and globally [60]. Proline is an amino acid that functions as an osmoregulator, protecting membranes and enzymes, and scavenging free radicals to support plant growth during osmotic stress [61]. Under the 96 h exposure time, the ATZ and Gly groups in the G1 (100 μg L^−1^) period exhibited a notable increase in their proline, fatty acid, and lipase contents. The reason for this could be that proline enhances plants’ ability to withstand adverse conditions by boosting the levels of fatty acids, soluble proteins, and other osmoregulatory chemicals in response to stress [62]. Several studies have demonstrated that certain microalgal biomass can contain up to 75% lipid content [63].

Fatty acids are the primary lipid metabolites and are categorized into saturated fatty acids (SFAs), monounsaturated fatty acids (MUFAs), and polyunsaturated fatty acids (PUFAs) [64]. Saturated fatty acids (SFAs) comprise the predominant fatty acids, constituting 83–93% of the overall composition [65]. Fatty acid synthesis is intricately linked to chloroplasts, which serve as the primary location for acetyl coenzyme biosynthesis. Acidification is an essential component for the creation of new fatty acids [66]. Fatty acids have a crucial role in the structure of cell membranes, and an increase in their concentration suggests that algal cells are trying to enhance the stability of their cell membranes in response to external pressures [67]. Lipase is a hydrolyzing oil esterase enzyme that mostly acts on virgin oil. It specifically targets the ester bond that links fatty acids and glycerol in the oil [68]. The current study observed that the alterations in fatty acid composition were not uniform among the different treatment groups and time intervals, possibly due to variations in the kind and concentration of the chemicals used, as well as the duration of exposure. For instance, ATZ exhibited a statistically significant bigger rise in *Navicula* during the G1 (100 μg L^−1^) compared to the G2 (300 μg L^−1^) treatment, whereas Gly demonstrated a more pronounced decline in *Navicula* during the G2 treatment. Multiple aspects must be considered when assessing the impact of these chemicals on aquatic ecosystems. These elements include the specific type of chemical, its concentration, the duration of exposure, and the species and physiological condition of the algae.

## 4. Materials and Methods

### 4.1. Chemicals and Reagents

Atrazine (ATZ), with the chemical formula C_8_H_14_CIN_5_ and a purity level exceeding 98.5%, was acquired from Zhancheng (Tianjin) Technology Co., Ltd., located in Tianjin, China. Glyphosate (Gly) with a chemical formula of C_3_H_8_NO_5_P and a purity level exceeding 95%, was acquired from Shanghai McLean Biochemical Co., Ltd., located in Shanghai, China. The chemical compound acetochlor (ACT) with the chemical formula C_14_H_20_CINO_2_, which is an analytical standard, was acquired from Shanghai Aladdin Biochemical Technology Co., Ltd., located in Shanghai, China. Chemical formulae, molecular structures, and weights are presented in Table 1.

### 4.2. Algae Growth and Cultivation

The *Navicula* sp. (MASCC-0035) was acquired from the microalgae species and quality resource bank of Seaweed Culture Collection Centre, Institute of Oceanology, Chinese Academy of Sciences (Qingdao, China). The microalgal strains were cultivated in F/2 medium at a temperature of 24 ± 2 °C, with an illumination intensity of 2000 Lx and a light-to-darkness ratio of 12 h of light followed by 12 h of darkness. The duration of the experiment was 96 h, during which the *Navicula* algae were acclimated and cultivated for a period of 14 days prior to the formal commencement of the experiment.

### 4.3. Algal Toxicity

The exposure studies were conducted using a series of 1 L glass beakers with F/2 medium. The experiment was partitioned into three experimental groups, each consisting of two concentrations, and each group was replicated three times. The experimental groups were as follows: (1) G1: atrazine (ATZ) powder was added to F/2 medium at concentrations of 100 µg L^−1^ and 300 µg L^−1^ followed by Du et al. (2023) [69]; (2) G2: glyphosate (Gly) powder was added to F/2 medium at concentrations of 100 µg L^−1^ and 300 µg L^−1^ following Lin et al. (2023) [70] and (3) acetochlor (ACT) powder was added to F/2 medium at concentrations of 100 µg L^−1^ and 300 µg L^−1^ following Liu et al. (2023) [7]. The control group consisted of the pure culture without any herbicides. Throughout the culture phase, the beaker was agitated at regular intervals of three times per day to guarantee the ideal development pattern of *Navicula*. The algae were cultivated for a duration of 96 h (4 days) under identical circumstances to those used during the pre-domestication phase. The cultures were obtained at time intervals of 0, 24, 48, 72, and 96 h, and the physiological and biochemical parameters were measured. The cell density was quantified using an ultraviolet spectrophotometer at the conclusion of the experiment. Details on the applied treatments are presented in Table 2.

### 4.4. Measurement of Cell Density

Initially, the concentrated algal solution was diluted into several density gradients using a medium. Subsequently, a hemocytometer measuring 22 × 26 × 0.5 mm was employed with the aid of an optical microscope to determine the concentration of algal cells in each gradient solution (averaging three counts). The algal solution’s absorbance was quantified at a wavelength of 680 nm using a UV-vis spectrophotometer. Through thorough data analysis, a linear correlation between the number of algal cells and the optical density at 680 nm (*OD*_680_) was determined [71].

$$\mathrm{Cell}\;\mathrm{numbers}\;\mathrm{of}\;Navicula\;\mathrm{sp}.\;\mathrm{cells}\;{\mathrm{mL}}^{-1}=8\times 10^{6}{OD}_{680}-10^{7}(R^{2}=0.9973)$$

### 4.5. Quantification of Pigment Concentration

The chlorophyll determination method was based on the approach described by Zhang et al. (2019) [72]. Briefly, *Navicula*, weighing approximately 1 g, was pulverized using liquid nitrogen, then dissolved in nine times its volume of pH 7.4 phosphate-buffered brine. The resulting mixture was then centrifuged in an ice bath at a speed of 5000 rpm for 15 min. The levels of chlorophyll a and chlorophyll b in the liquid portion were measured following the directions provided with the kit (Jiangsu Enzyme Free Industrial Co., Ltd., Yancheng, China).

### 4.6. Assessment of the Antioxidative Activities

In order to gain a deeper understanding of how *Navicula* responds to the toxic effects of three common herbicides, this study examined the impact of these herbicides on the *Navicula* antioxidant defense system. Specifically, the study assessed the activities of superoxide dismutase (SOD), catalase (CAT), and peroxidase (POD), as well as the levels of malondialdehyde (MDA) and reactive oxygen species (ROS) in *Navicula*. *Navicula* samples were obtained at concentrations of 100 μg L^−1^ and 300 μg L^−1^ herbicides at 0, 24, 48, 72, and 96 h following treatment. Each group underwent three repetitions. Afterwards, the level of oxidative damage in *Navicula* was evaluated using a commercially accessible kit (Jiangsu Meimian Industrial Co., Ltd., Yancheng, China) in accordance with the instructions provided by the manufacturer.

To examine the possible mechanisms behind the effects of ATZ, ACT, and Gly on the growth of microalgae in *Navicula*, we assessed the impact of these three herbicides on various osmoregulatory substances in microalgae, including soluble sugars, soluble proteins, free proline, fatty acids, and phytolipases. Algae samples were obtained at time intervals of 24, 48, 72, and 96 h following the treatment. The repetition of each group occurred three times. Afterwards, we assessed the osmoregulatory compounds of *Navicula* utilizing a commercially accessible kit (Jiangsu Meimian Industrial Co. Ltd., Yancheng, China) in accordance with the instructions provided by the manufacturer.

### 4.7. Statistical Analysis

The toxicity test data were subjected to statistical analysis using Origin^®^10.0 (Origin lab Corporation, Northampton, MA, USA) and Statistix 8.1. The results are expressed as the mean value ± standard deviation (SD). The statistical analysis involved the use of single-component analysis of variance (ANOVA) and Tukey multiple-comparison tests, with a significance level set at *p* < 0.05.

## 5. Conclusions

The experimental data showed that there were significant differences in the responses of the three herbicides. Atrazine and glyphosate exhibited greater sensitivity to *Navicula* compared to etofenprox, possibly because of variations in cell wall composition and cell size. Nevertheless, this distinction may not always be evident, as algal reactions are also affected by various additional parameters like temperature, light, nutrient concentrations, and other environmental pressures. Furthermore, it is crucial to focus on the processes of pesticide bioaccumulation and transformation in aquatic ecosystems and their enduring impacts on the composition and variety of aquatic communities. Hence, it is imperative to conduct a more comprehensive investigation into the interplay of these elements to gain a deeper comprehension of the deleterious impacts of herbicides on algae.

## Figures and Tables

**Table 1:** Basic attributes of herbicides used in the study.

Compound	Molecular Formula	Molecular Structure	Molecular Weight
Atrazine	C_8_H_14_CIN_5_	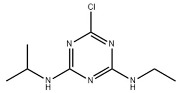	215.68
Glyphosate	C_3_H_8_NO_5_P	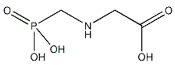	169.07
Acetochlor	C_14_H_20_CINO_2_	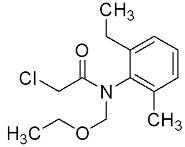	269.77

**Table 2:** Treatments and concentrations used in the study.

Serial Number	Treatments	Description
1	CK	Control
2	ATZ-G1	Atrazine 100 µg L^−1^
3	ATZ-G2	Atrazine 300 µg L^−1^
4	Gly-G1	Glyphosate 100 µg L^−1^
5	Gly-G2	Glyphosate 300 µg L^−1^
6	ACT-G1	Acetochlor 100 µg L^−1^
7	ACT-G2	Acetochlor 300 µg L^−1^

## Data Availability

Data will be available on request.

## Abbreviations

The following abbreviations are used in this manuscript:

**Serial Number**	**Abbreviation**	**Details**
1	ATZ	atrazine
2	ACT	acetochlor
3	Gly	glyphosate
4	EPA	Environmental Protection Agency
5	EC	European Commission
6	Chlla	chlorophyll a
7	Chllb	chlorophyll b
8	SS	soluble sugar
9	SP	soluble protein
10	ROS	reactive oxygen species
11	MDA	malondialdehyde
12	SOD	superoxide dismutase
13	CAT	catalase
14	POD	peroxidase
15	FP	free proline
16	FA	fatty acid
17	SFAs	saturated fatty acids
18	MUFAs	monounsaturated fatty acids
19	PUFAs	polyunsaturated fatty acids
